# Current Systemic Treatment Options in Metastatic Urothelial Carcinoma after Progression on Checkpoint Inhibition Therapy—A Systemic Review Combined with Single-Group Meta-Analysis of Three Studies Testing Enfortumab Vedotin

**DOI:** 10.3390/cancers13133206

**Published:** 2021-06-26

**Authors:** Susanne Deininger, Peter Törzsök, David Oswald, Lukas Lusuardi

**Affiliations:** Department of Urology and Andrology, Salzburg University Hospital, Paracelsus Medical University, 5020 Salzburg, Austria; p.toerzsoek@salk.at (P.T.); d.oswald@salk.at (D.O.); l.lusuardi@salk.at (L.L.)

**Keywords:** urothelial carcinoma, metastases, checkpoint inhibition therapy, antibody drug conjugate, target therapy

## Abstract

**Simple Summary:**

Currently, little is known about what therapeutic options exist for the treatment of metastatic urothelial carcinoma (mUC) after a therapy with checkpoint inhibitors (CPI). In the context of this systemic review, five therapy regimens tested in the post-CPI setting with adequate data were identified: Chemotherapy (CT), Ramucirumab plus Docetaxel, Erdafitinib (Erd), Enfortumab vedotin (EV), and Sacituzumab govitecan (SG). Most data were available on EV, and the results of three studies testing the agent were combined via single-group meta-analysis. For EV, the objective response rate was 42.1% compared to 17.9% for CT in a similar setting. EV was also ahead in progression-free survival (5.9 months with EV vs. 3.7 months with CT) and overall survival (12.8 months with EV vs. 9.0 months with CT). Further research is needed on the question of which patients’ subcollectives particularly benefit from which therapeutic approach.

**Abstract:**

Background: In the first and second-line therapy of metastatic urothelial carcinoma (mUC), checkpoint inhibitors (CPI) such as Pembrolizumab and Atezolizumab have been widely implemented. Little is currently known about what therapeutic options are effective after therapy with CPI. This article presents a systemic review of current treatment options in this setting. Methods: From August 2020 to 15 April 2021, a literature search was performed through the PubMed/Medline. Subsequently, a single-group meta-analysis of three studies testing Enfortumab vedotin (EV) was conducted. Results: Five therapy regimens tested in the post-CPI setting with adequate data were identified: Chemotherapy (CT), Ramucirumab plus Docetaxel, Erdafitinib (Erd), EV, and Sacituzumab govitecan (SG). In *n* = 74 + 125 + 288 patients, the single-group meta-analysis showed an objective response rate of 42.1% for EV compared to 17.9% for CT in a similar setting. EV was also ahead in progression free survival (5.9 months with EV vs. 3.7 months with CT) and overall survival (12.8 months with EV vs. 9.0 months with CT). Conclusion: Most data are currently available for EV. Further research is needed on the question of which patients’ subcollectives particularly benefit from which therapeutic approach.

## 1. Introduction

According to the latest global cancer statistics, bladder cancer (BC) was the 10th most common cancer worldwide in 2020. Among men, BC is in 6th place [1]. The most important risk factor for the development of BC is smoking (50%) [2], where the risk correlates with the length of abuse and the amount of cigarettes consumed [3]. Contact with chemicals like aromatic amines in the course of occupational activities is considered to be the cause of 10% of BC [4]. Environmental factors such as exposure to ionizing radiation [5], arsenic [6] and consumption of chlorinated drinking water [7] are also associated with increased BC risk.

With about 75% of cases, pure urothelial carcinoma (UC) is the most common histological form of BC [8]. Besides, there are UCs with divergent differentiations, such as squamous, neuroendocrine, micropapillary, sarcomatoid, and others. Non-UC dignities such as small cell cancer are rare and mostly aggressive [9].

In the last few years, a lot has happened in pathological and genetic UC research. It has been shown that UC differs greatly in molecular biology and gene expression. In 2014, the Cancer Genome Atlas (TCGA) project for urothelial bladder cancer provided a detailed molecular genetic map of 131 high grade UCs. Thirty-two relevant recurrent mutations were identified. Potential targets for individualized therapies were identified in 69% of the tumors, including 42% in the phosphatidylinositol-3-OH kinase/protein kinase B (AKT)/mechanistic target of rapamycin (mTOR) pathway and 45% in the receptor tyrosine kinases (RTK)/mitogen-activated protein kinase (MAPK) pathway [10]. Later, the following subtyping was implemented based on the molecular features: luminal-papillary (cluster I), luminal-nonspecified, luminal unstable, stroma-rich, basal/squamous and neuroendocrine-like [11,12].

BC is classified according to TNM [13] like other tumor entities, but is additionally subdivided in non- muscle invasive BC (NMIBC, up to pT1) and muscle invasive BC (from pT2). At the time of first diagnosis, up to 86% of tumors were classified as NMIBC [14,15]. The depth of the tumor invasion and the tumor grading have decisive influences on the therapy and prognosis.

Transurethral resection of the bladder is usually the method of choice for histologic confirmation of the tumor and may also be the definitive treatment in some stages of NMIBC. Based on histological and clinical features NMIBC is divided into risk classes [16] predicting the risk of recurrence and progression. Risk factors include number of tumors, tumor size, prior recurrence rate, T category and concomitant carcinoma in situ and grading. Bladder instillation therapies with, for example, Bacillus Calmette-Guérin and Mitomycin C can reduce the risk of recurrence and/or progression in certain intermediate and high-risk constellations [17,18]. Depending on the risk profile, 1–45% of primary tumor NMIBC progress to MIBC [19]. If muscle invasion is detected, there is an indication for cystectomy combined with pelvic lymphadenectomy and urinary diversion. In this case, the administration of a neoadjuvant CT may improve five-year overall survival (OS) by 5% [20].

A metastatic stage, primary or secondary, is present in 5% of UC patients, and five- year OS in this setting is only 4.6% [21]. The systemic therapy in metastatic UC (mUC) has undergone a true revolution in the last few years. First-line CT with methotrexate, vinblastine, Adriamycin and Cisplatin (M-VAC) was the standard of care since 1988 [22]. In the year 2000, a new form of first-line CT emerged and showed promising results: platinum-based CT (mostly Cisplatin) in combination with Gemcitabine (GemCis). The results in terms of OS, objective response rate (ORR) and duration of response were comparable to M-VAC, but the GemCis therapy regime was better tolerated. Overall, there were fewer severe therapy-associated adverse events (AEs) such as (febrile) neutropenia, and the therapy lines could be given more frequently at full dosage with GemCis [23]. For a long time, the only approved second-line therapy in the case of tumor progression has been Vinflunine [24]. In combination with best supportive care (BSC) it provided an improvement of median OS of 2.6 months compared to BSC alone (*p* = 0.04) [25,26]. Other second-line CT regimens like Docetaxel, Taxanes or Cyclophosphamide, were less established.

From 2016, the next therapy reform was on the horizon: checkpoint inhibition (CPI) therapy. Atezolizumab (Tecentriq^®^) was the first programmed cell death ligand-1 (PD-L1)-antibody to be approved by the United States Food and Drug Administration (FDA) [27,28] as a second-line therapy after platinum-based CT. Later, the indication was extended to first-line therapy in platinum-ineligible patients according to the IMvigor210 study [29]. Soon after, the PD-1 antibody Pembrolizumab (Keytruda^®^) was approved in the same indications [30,31]. In total, the following CPIs are FDA approved for the treatment of mUC in second-line therapy after platinum-based CT: Pembrolizumab, Atezolizumab, Durvalumab, Nivolumab and Avelumab.

However, the problems of CPI soon became clear: only about one-quarter to one-fifth of patients respond to CPI. In the first-line setting in Cisplatin-ineligible patients Pembrolizumab showed an ORR of 28.6% in the Keynote-052 study. The ORR was higher in patients with Combined Positive Score (CPS) higher than or equal to 10 (47.3% vs. 20.3% in patients with CPS < 10). Other factors, like visceral metastases, also influenced the objective response rate (ORR). A hitherto almost unknown phenomenon was revealed: 8.9% of patients had no detectable disease during ongoing therapy (=complete response (CR)). The median duration of response (DoR) was 30.1 months. Still, it should not be forgotten that in the Keynote-052 study, 42.4% of Cisplatin-ineligible patients under Pembrolizumab in the first line setting achieved progressive disease (PD) as the best response [32]. A similar picture emerged with the use of CPI in second-line therapy after progression under platinum-based CT: ORR was only around 20% [28,31]. Even if a primary response had occurred, only a small minority would respond permanently to CPI. The median OS under CPI therapy was 10–16 months [31,33] depending on the setting.

In the next few years, a new group of patients will emerge with PD on CPI therapy. Assuming they are in good general health, these patients will need established treatment options in the future.

The following article reviews the available systemic therapies studied in the patient collective with mUC after prior CPI therapy. In the article, mUC is used as an umbrella term for mUC or locally advanced and unresectable UC (i.e., Stage III or IV disease).

## 2. Materials and Methods

### 2.1. Literature Search

From August 2020 to 15 April 2021, a literature search was performed by the authors through the PubMed/Medline to identify available studies of systemic therapeutic options that have been investigated in a subcollective with mUC patients after prior CPI therapy. The search was performed using the following search terms in different combinations: “bladder cancer”, “antibody drug conjugate”, “Checkpoint inhibition”, “metastatic urothelial carcinoma”, “after”, “bladder cancer”, “Sacituzumab govitecan”, “Erdafitinib”, “Enfortumab vedotin”, “target therapy” and “trial”. The reference lists of the studies found were also used to obtain additional relevant literature. Current abstracts with preliminary data from international congresses, such as the Genitourinary Cancers Symposium of the American Society of Clinical Oncology (ASCO-GU) and the European Association of Urology (EAU), were reviewed with regard to the research question, and partly included in the text.

### 2.2. Study Selection

**Figure 1:**
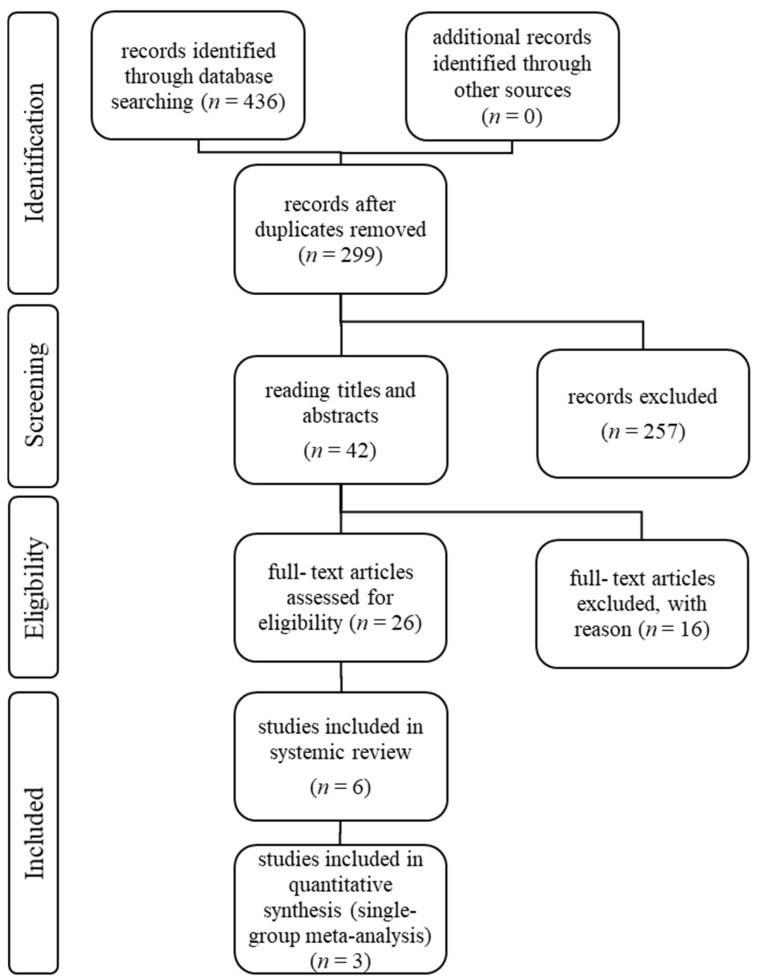
An overview over the study selection following the recommendations of the PRISMA-Statement.

The authors performed the study selection following the recommendations of the Preferred Reporting Items for Systematic Review and Meta-analysis Statement (PRISMA) [34]. The study selection process can be seen in flowchart Figure 1. Only publications that evaluated a subgroup with mUC patients after prior CPI therapy written in English were included in text and tables. Duplicated studies were excluded. All included publications described prospective, multicenter pharmaceutical studies, with one or more treatment arms. Case reports were excluded. One retrospective data analysis was included in the text, but not in the tables. No other retrospective analyses were included. The abstracts of the publications were checked for eligibility and, if the criteria were met, the entire article was reviewed.

### 2.3. Data Extraction

Data extraction was performed by the authors. A uniform data table was used, which contained the following information: general information of the publication (name, authors, journal and year of publication), type of study, study phase, details concerning randomization, baseline characteristics of the patients (number, mean value and standard deviation of age, sex, Eastern Cooperative Oncology Group (ECOG), sites of metastasis (lymph nodes only or visceral including liver, lungs, bones), prior therapies including cis-/carboplatin base CT, or CPI, and outcome parameters including CR, PR, SD, PD, ORR, PFS, OS, DoR in the subgroup after prior CPI therapy. In all studies included in the systemic review, response rates were determined using response evaluation criteria in solid tumors (RECIST) version 1.1 [35]. Adverse events (AEs) were analyzed independently of the subgroup of CPI pretreated patients, as these data were mostly only available for the total collective of included patients. The authors assumed that they were representative for the AEs in the subgroup of CPI pretreated patients.

### 2.4. Statistical Methods

Three consecutive studies were identified which, despite the different study phases, investigated EV in a similar study design in a comparable patient cohort at the same dosage of EV. Thus, a single group meta-analysis was created to describe the outcome of EV. The effect size of proportions was estimated using a single group meta-analysis based on the inverse variance method. For proportions, exact Clopper-Pearson confidence intervals are presented. Based on the estimated heterogeneity between the three studies, which was assessed using Higgins I^2^ and Cochran’s Q, a fixed effect or random effects model was estimated. The effect size (median time-to-event) of time-to-event variables (DoR, PFS, OS) was estimated based on the Kaplan-Meier estimators. Thus, the accuracy of the calculated estimators is limited. Descriptive parameters were described as pooled results using the corresponding study sample size as weight.

A *p*-value < 0.05 was taken as the uncorrected statistical significance level (two-sided); therefore, all inferential results are only descriptive. For statistical analysis, the statistical computing software R Version 4.0.3 (R Foundation for Statistical Computing, Vienna, Austria) was used. For conducting the meta-analysis, the R packages meta [36], metamedian [37] and MetaSurv [38] were used.

A presentation of selected statistical calculations of the single group meta-analysis can be found as Supplementary Material File S1.

## 3. Results

The demographic data of the patients’ collectives of the selected studies can be found in Table 1. The outcome parameters of the subgroups of mUC patients after prior CPI therapy are depicted in Table 2. The safety profile and the most common adverse events (AEs) of the study drugs in the patients’ collectives, independent of prior CPI, defined as AEs of all grades in ≥20% of patients or ≥grade 3 (G3) AEs in ≥5% of patients, can be found in Table 3. In all studies included in the systemic review, the AEs were classified according to Common Terminology Criteria for Adverse Events (CTCAE) [39].

### 3.1. Chemotherapy (CT) Alone or as Combination Therapy

#### 3.1.1. Gemcitabine Plus Cisplatin or Carboplatin, Docetaxel, Paclitaxel, Vinflunine and Others

For a long time, CT was the only treatment option for mUC in the first and subsequent lines. After progression on platinum-based CT, mostly Vinflunine was used [24]. Still, CTs like docetaxel [43] and paclitaxel [47] were also available. With the advent of CPIs, the question arose as to whether these chemotherapy regimens could be useful further down the sequence, after progression on CPI therapy. The phase 3 EV-301 study [42] compared the antibody-drug conjugate (ADC) Enfortumab vedotin (EV) (see separate data below) with CT with docetaxel, paclitaxel, or Vinflunine for mUC after prior platinum-based CT and CPI therapy. The patients were randomized in a 1:1 ratio. The CT regimen was investigator-choice, but a maximum of 35.0% of study participants was allowed to receive Vinflunine. Out of 307 patients assigned to receive CT, one-hundred seventeen (38.1%) were assigned to receive docetaxel, one-hundred and twelve (36.5%) paclitaxel, and seventy-eight (25.4%) Vinflunine, respectively. The response to previous CPI was “response” in 16.5% patients and “no response” in 70.0%. Two hundred ninety-one participants (94.8%) received at least one dose of the study drug.

The median OS in the CT collective was 9.0 months (95% CI, 8.1–10.7), and the median PFS 3.7 months (95% CI, 3.5–3.9). The ORR was 17.9% (95% CI, 13.7–22.8), with 2.7% CR. The DoR was 8.1 months.

Of the patients, 91.8% experienced at least one AE, with 49.8% ≥G3 AEs. The most common AEs in the CT regimen group were alopecia (36.4%), peripheral sensory neuropathy (PSN, 21.3%), decreased appetite (23.4%), nausea (21.6%), and anemia (20.3%). The most common ≥G3 AEs were decreased neutrophil count (13.4%), anemia (7.6%), and decreased white blood cell (WBC) count (6.9%). Due to AEs, dose modifications were necessary in 27.5%, and the treatment was terminated in 11.3%.

In 2020, Gomez de Liano Lista A. et al. published their experience on chemotherapy vs. BSC in the post CPI setting in the form of a retrospective data analysis (data not shown in tables) [48]. Two hundred and seventy patients with mUC after prior CPI therapy were analyzed, sixty-nine after CPI in the first-line setting due to platinum-ineligible status (group 1), and two hundred and one after CPI therapy in the second-line after platinum-based CT (group 2). The applied CTs were Gemcitabine plus Cisplatin or Carboplatin, Taxanes, or miscellaneous/unknown. In group 1, *n* = 39 received a subsequent CT, whereas *n* = 30 went over to BSC. In group 2, *n* = 68 received a subsequent CT, whereas *n* = 133 went over to BSC.

The ORR of CT was 58.0% for group 1 (*n* = 33), and 31.0% for group 2 (*n* = 54). The ORR did not differ between the agents (Cisplatin or Carboplatin vs. others). The median PFS was 5.6 months for group 1, and 3.8 months for group 2. The OS was 6.8 months for group 1 compared to 1.9 months for BSC in the same setting, and 8.3 months for group 2 compared to 1.5 months for BSC in the same setting. In group 2, response to CPI therapy (*p* = 0.03), length of CPI therapy (*p* = 0.002) and the receipt of CT (*p* < 0.001) were associated with longer OS. Despite the retrospective design, the response and survival data appear similar to those of the EV-301 study.

#### 3.1.2. Docetaxel Plus Placebo or Ramucirumab (RAM)—An Epidermal Growth Factor Receptor (EGFR) Antibody—The RANGE Study

In 2017, the data from the RANGE study was published [43,49]. It was a phase 3 study which included 530 patients with mUC after prior platinum-based CT. Previous treatment with one CPI was allowed if the platinum-based CT had not been finished longer than 24 months ago.

The collective was randomized in a 1:1 ratio to receive intravenous (i.v.) Docetaxel 75 mg/m^2^ (Taxotere^®^) plus either RAM 10 mg/kg (D + R) or placebo (D + P) on day one of a 21-day cycle.

Two hundred sixty-three patients were assigned to D + R, while two hundred sixty-seven patients were assigned to D + P.

The general collective showed an ORR 25.9% for D + R and 13.9% for D + P. The median DoR was 5.3 months for D + R and 4.2 for D + P (*p* = 0.19). Median PFS after sensitivity analysis was 4.1 months (95% CI 3.3–4.8) for D + R vs. 2.8 months (95% CI 2.6–2.9) for D + P (*p* = 0.0002). Median OS was 9.4 months (95% CI 7.9–11.4) for D + R and 7.9 months for D + P (stratified HR 0.887 (95% CI 0.7–1.1); *p* = 0.25). So, the study failed to show improvement of OS by adding RAM to docetaxel [43].

Forty-five individuals out of the intention to treat (ITT) population (*n* = 530) had received prior CPI (Atezolizumab in 46.7%, Pembrolizumab in 33.3%, Durvalumab ± Tremelimumab in 8.9%, Nivolumab in 6.7% and Bgba317 (anti-PD-1) in 4.4%). Seventeen patients with prior CPI therapy were assigned to receive D + R, and twenty-eight D + P. In the subgroup of CPI pretreated patients, the ORR was 29.4% for D + R and 7.1% for D + P. Thirty-five percent of patients with D + R, and 57.1% with D + P achieved SD, whereas 17.6% with D + R and 25.0% with D + P achieved a best response of PD. The data for PFS and OS did not differ between the groups but was not shown in the publication [43,50].

Two hundred and fifty-eight patients under D + R and two hundred and sixty-five patients under D + P were available for the safety population analysis. Treatment related AEs were reported in 85.7% of patients under D + R, and in 84.2% of patients under D + P. The most common AEs were fatigue (39.1% for D + R and 36.2% for D + P), alopecia (23.6% for D + R and 30.6% for D + P) and diarrhea (23.6% for D + R and 16.6% for D + P). At least G3 AEs were reported in 47.7% of patients under D + R, and in 40.8% under D + P. The most common ≥G3 AEs were hematological: neutrophil count decrease (8.9% for D + R and 10.2% for D + P) and febrile neutropenia (9.3% for D + R and 6.0% for D + P. Treatment was discontinued due to AEs in 19.4% (D + R) and 7.5% (D + P).

Clinical use and outlook: RAM (Cyramza^TM^) is FDA-approved in the following indications: metastatic non- small- cell lung cancer and gastrointestinal cancers (gastric, gastroesophageal junction, colorectal) alone or in combination with CT. There is no approval in UC.

### 3.2. EV-an ADC Targeting Nectin-4

#### 3.2.1. The EV-101, EV-201 and EV-301 Studies—Study Design

EV as an Antibody-Drug-Conjugate (ADC) targets the calcium-independent cell-cell adhesion molecules Cadherin Nectin-4 [51], mediating immunomodulation. The studies EV-101, -201 and -301 are consecutive clinical studies that have a comparable study design with the same dosage and application modalities of EV at least in one subgroup. Therefore, the authors decided to pool the demographic data of the patients and to perform a statistical analysis (single-group meta-analysis) of outcome and survival parameters, as well as frequency of AEs. The data can be found in the Table 1, Table 2 and Table 3.

The EV-101 study was a phase 1 study evaluating EV in mUC patients after either prior systemic therapy or in platinum-ineligible condition. The dose escalation phase provided increasing doses of EV (0.5, 0.75, 1.0, and 1.25 mg/kg of body weight), as i.v. infusion on days 1, 8 and 15 of a 28-day cycle. One hundred and fifty-five mUC patients were included in the study. One hundred and twelve patients received a dosage of 1.25 mg EV/kg of body weight, which was later established as the standard dosage. Out of these 112 patients, 74 had received prior CPI therapy and were available for central review.

Due to promising outcome data, the phase 2 confirmatory study EV-201 was launched [41]. The study included patients with mUC after prior platinum-based CT and CPI therapy. The study participants received 1.25 mg EV/kg of body weight i.v. on days 1, 8 and 15 of a 28-day cycle. One hundred and twenty-five patients were included. The responses to prior CPI therapy were “response” in 20% and “no response” in 80%.

The data from the subsequent phase 3 clinical study EV-301 has just been published [42]. It compared the use of EV with CT with docetaxel, paclitaxel, or Vinflunine for patients with mUC after prior platinum-based CT and CPI therapy. The patients were randomized in a 1:1 ratio. The CT regimen was investigator-choice (see separate data above). Three hundred and one patients received 1.25 mg EV/kg of body weight i.v. days 1, 8 and 15 of a 28-day cycle.

#### 3.2.2. Study Data

The response data of 487 patients (*n* = 74 [40] + 125 [41] + 288 [42]) were analyzed via single-group meta-analysis. The ORR was 42.1% (95% CI, 37.8–46.5), with 8.5% (95% CI, 4.6–15.1) CR and 34.5% (95% CI, 30.4–38.9) PR. Of the patients, 31.3%achieved best response of SD (95% CI, 27.3–35.5) and 16.7% experienced PD (95% CI, 13.6–20.3).

The parameters DoR, PFS and OS were extracted using the available Kaplan-Meier estimators.

The median DoR was 7.5 months (*n* = 33 [40] + 55 [41] + 117 [42]), the median PFS was 5.9 months (95% CI, 5.4–6.6) (*n* = 74 [40] + 125 [41] + 301 [42]), and the OS was 12.8 months (*n* = 89 [40] + 125 [41] + 301 [42]).

A total of 93.8% of patients (95% CI, 91.1–95.8) experienced any grade of treatment-related AEs. The most common AEs were fatigue (43.9% (95% CI, 29.8–58.9)), PSN (36.1% (95% CI, 32.1–40.2)), concerning the gastrointestinal tract (decreased appetite (38.3% (95% CI, 29.6–47.7)), dysgeusia (33.5% (95% CI, 23.4–45.4)), nausea (32.3% (95% CI, 21.8–45.0)), diarrhea (28.1% (95% CI, 24.5; 32.1)) or the skin or hair (alopecia (46.3% (95% CI, 42.1–50.6)), pruritus (27.6% (95% CI, 18.7–38.7)), maculopapular rash (20.9% (95% CI, 15.2–27.9)). At least G3 AEs occurred in 52.3% (95% CI, 47.5–57.0) of patients. The most common ≥G3 AEs were neutrophil count decrease (6.7% (95% CI, 4.7–9.5)), maculopapular rash (6.0% (95% CI, 4.3–8.5)), and fatigue (5.7% (95% CI, 3.9–8.1)). An AE of special interest mentioned in the EV-101 and -201 studies was hyperglycemia, which de novo occurred in 5–11% of patients [40,41], but was no longer mentioned in the EV-301 study.

### 3.3. Clinical Use and Outlook

The FDA granted accelerated approval to EV (PADCEV^©^) for patients with mUC after prior platinum-based CT and CPI therapy on December 2019 [52]. According to the data from the recently published EV-301 study [42], the European Medicines Agency (EMA) announced accelerated assessment for EV [53].

### 3.4. Erdafitinib (Erd)—A Pan-Fibroblast Growth Factor Receptor (FGFR) Tyrosine Kinase Inhibitor

#### 3.4.1. The BLC2001 Study—Study Design and Results

In 2019, Loriot et al. published the data of the BLC2001 study with the pan-FGFR Tyrosine Kinase Inhibitor Erd [44]. The study enrolled patients with mUC with certain FGFR2 or -3 mutations. All patients had received at least one prior CT in a metastatic setting or experienced progression within 12 months after neoadjuvant or adjuvant CT. Initially, two different dosage regimens were used: 10 mg daily for one week followed by one week off vs. 6 mg daily continuous. After an interim analysis, the continuous administration of 8 mg (with permitted dose escalation to 9 mg daily) was chosen to be selected regimen (SRM). Ninety-nine patients were assigned to the SRM. The collective had the following outcome parameters: ORR 40.4% (95% CI, 31.0–50.0), with 3.0% CR and 37.4% PR. Of the patients, 39.4% achieved SD and 18.1% PD. The median PFS was 5.5 months (95% CI, 4.2–6.0), the median OS 13.8 months (95% CI, 9.8–not reached) and the median DoR 5.6 months (95% CI, 4.2 to 7.2).

Twenty-one patients of the collective had received CPI therapy before study inclusion. The subgroup of these patients was evaluated only with respect to ORR, which was 59%. Only 5% (*n* = 1) of these patients had previously responded to CPI according to the study coordinators’ analysis.

The most common AEs in the SRM collective were hyperphosphatemia (76.8%), stomatitis (57.6%), and diarrhea (50.5%). At least G3 AEs were reported in 46.0% of patients. The most common ≥G3 AEs were hyponatremia (11.1%), stomatitis (10.1%) and asthenia (7.1%). Thirteen percent of patients discontinued therapy due to AEs, including central serous retinopathy and skin and mucosa related AEs.

#### 3.4.2. Clinical Use and Outlook

In December 2019 the FDA granted accelerated approval of Erd (BALVERSA^TM^) in the following indication [54]: mUC with FGFR3 or FGFR2 GA within 12 months after at least one line of prior platinum-based CT (adjuvant or neoadjuvant) [55]. There is no approval from the EMA.

A phase 3 study is ongoing comparing Erd with CT with Vinflunine or Docetaxel or Pembrolizumab in patients with mUC with selected FGFR GA in the second or third-line setting [56].

### 3.5. Sacituzumab Govitecan (SG)—An ADC Consisting of an Immunglobulin G (IgG) Antibody Targeting Troponin-2 and the Topoisomerase-I Inhibitor SN-38

#### 3.5.1. The IMMU-132 and the TROPHY-U-01 (IMMU-132–06) Studies—Study Design and Results

The first data of the phase 1 basket study IMMU-132 was published in 2017. As part of the study patients with diverse metastatic cancers received 8 or 10 mg/kg SG on days 1 and 8 of a 21-days cycle [45]. Ninety-seven patients with different tumor entities received a SG dosage of 10 mg/kg of body weight, which would later be set as the standard dose.

Part of the data concerning the outcomes of 45 mUC patients was published separately in the form of an abstract [46]. Thirty-eight percent of patients had received prior CPI before study inclusion. The ORR was 31.1% in the complete mUC collective, and 23.5% in the CPI pretreated patients. The median DOR was 12.6 months, the median PFS was 7.3 months, and the median OS was 18.9 months.

Of the patients, 91.8% experienced treatment related AEs (*n* = 89). The most common AEs were diarrhea (56.7%), nausea (57.7%), fatigue (47.4%) and neutrophil count decrease (53.6%). The most common ≥G3 AEs were neutrophil count decrease (33.0%), WBC count decrease (11.3%) and anemia (11.3%).

The design of the still ongoing phase 2 TROPHY-U-01 study consists of three treatment arms: patients with mUC after prior therapy with CPI, platinum-based CT, or both shall receive SG [57,58]. The first data of the patient cohort after prior CT and CPI therapy was published at the 2020 European Society for Medical Oncology (ESMO) virtual congress.

One hundred and thirteen patients were included. The ORR was 27%, with a CR rate of 5%. The median DoR was 5.9 months. The median PFS was 5.4 months (95% CI 3.5–6.9), and the median OS was 10.5 months (95% CI 8.2–12.3).

#### 3.5.2. Clinical Use and Outlook

The TROPHY-U-01 study is still ongoing. A confirmatory (Phase 3) study investigating the anticancer activity of SG (Trodelvy^TM^) in mUC is the TROPICS-04 study [59] that started in august 2020. The study includes patients with mUC after prior platinum-based CT (in the neo-adjuvant or adjuvant setting) and CPI therapy. The design foresees a randomisation in a 1:1 ratio (SG vs. CT with Vinflunine (Javlor^®^), Paclitaxel (Abraxane©) or Docetaxel (Taxotere^®^)). Another clinical study that started in January 2021 tests SG plus EV in mUC after prior platinum-based CT and CPI therapy [60].

In April 2020, SG received accelerated FDA approval for the treatment of metastatic triple-negative breast cancer after at least two prior systemic therapies [61].

## 4. Discussion

CPI changed the world of therapy of mUC permanently. Now we increasingly need new therapeutic approaches to offer to future generations of patients. We still have the usual therapy option, which is CT, even though we know little about the response and survival data in this particular setting. The question arises whether CT in a setting after progress on CPI therapy, i.e., mostly in a third-line setting, makes sense. CT was tested in the third-line setting in pre-CPI times: In 2015, di Lorenzo et al. showed a median PFS of 3.3 and a median OS of 7.8 months in mUC patients treated with cyclophosphamide, platinum re-exposure, Vinflunine, Taxanes, or Gemcitabine in the third-line [62]. In the EV-301 study, CT achieved comparable outcomes after CPI with a median PFS of 3.7 months and a median OS of 9.0 months [42]; the same applied to the collective of Gomez de Liano Lista, et al. [48]. Compared to an OS of 1.5–1.9 months with BSC [48], this is a gain of several months, even if few of the patients benefited at all from CT. Only 18% of the patients in the EV-301 study showed any kind of response to CT. However, those who had previously benefited from the CPI seem to benefit from subsequent CT. A response to prior CPI therapy and the length of CPI was significantly associated with longer OS in the data of Gomez de Liano Lista, et al. [48].

SG as ADC containing SN-38, the active component of Irinotecan [63], represents a further development of classical CT. Irinotecan is a topoisomerase I inhibitor, which was never established in the treatment of mUC, neither alone [64] nor in combination with gemcitabine [65]. In the case of this ADC, the active drug is bound to an anti-Trop2 antibody directed against a protein frequently overexpressed in tumor cells [66]. It is not yet known how topoisomerase I antibodies interact with anti-PD-(L)1 antibodies. In animal models, the combination of Irinotecan with anti-PD-L1 antibodies has already been shown to have greater antitumor activity than either substance alone. Irinotecan and Anti-PD-L1 antibodies are thought to act synergistically: while Irinotecan reduces the density of regulatory T cells and increases tumor antigen presentation via major histocompatibility complex (MHC)-1, anti-PD-L1 antibodies block the likewise upregulated PD-L1 expression [67]. The combination therapy of the PD-L1 inhibitor Atezolizumab and SG will be investigated in a phase 1b/2 study in patients with mUC after platinum-based CT [68]. However, it is unclear whether the synergistic effect can also occur when CPI and SG are used in sequence. The ORR of patients with mUC after CPI therapy in the IMMU-132 study was only 23.5%, although the data are certainly of limited value given the small number of patients (*n* = 17) [46].

EV choses a different target than SG: Nectin-4, a calcium-independent cell-cell adhesion molecule cadherin [51], mediating immunomodulation. Most of the data related to our research questions exists on EV. Nectin-4 is moderately expressed in human skin and strongly expressed in tumor tissues, with an expression of >60% in UC [69]. In the EV-101 study almost all UC tumor biopsies had a high Nectin-4 expression [40], so that Nectin-4 expression was removed as a requirement for study inclusion. The patient collective analyzed in the EV-101, 201 and 301 studies with a total of *n* = 487, is the largest of the collectives mentioned in this article. The response rates of EV with 42.1% ORR and 8.5% CR are promising compared to CT in the comparable setting (ORR 17.9%, 2.7% CR). Median OS (12.8 months for EV vs. 9.0 months for CT) and PFS (5.9 months for EV vs. 3.7 months for CT) also favor EV, although comparability between studies is limited. The special focus of this article is aimed at the therapy of mUC after progression on CPI therapy. There is still controversy as to whether testing of PD-(L)1 status prior to initiation of therapy with a CPI is useful, since patients with low or absent expression also respond, as shown for example in the IMVigor 210 study [70]. We know less about any correlation between PD-(L)1 expression, response to immunotherapy, and response to subsequent therapies such as EV. The expression of Nectin-4, at least in upper tract urothelial carcinoma, does not seem to be correlated with the expression of PD-L1 as demonstrated by Tomiyama et al. on 99 tissue microarrays [71]. Another special patient group could benefit, in that very recent data shows that Nectin-4 expression is higher in luminal subtypes than in basal subtypes of UC (*p* < 0.01). The expression is also positively correlated with the luminal markers GATA3, FOXA1 and PPARG [72].

It has become apparent, that the molecular subtype of UC has an influence on the prognosis and response to therapies like CT [73], and presumably also CPI therapy [70]. Rosenberg et al. showed in 2016 that PD-L1 immune cell expression was higher in the basal compared to the luminal subtypes (*p* < 0.0001) of UC. Still, patients with the subtypes basal and luminal cluster I reached worse Atezolizumab response rates, indicating different pathways of immunosuppression than the PD-1/PD-L1 mechanism.

One point of approach in luminal subtypes could be FGFR inhibition, as they are particularly equipped with FGFR3 overexpression and activating FGFR3 mutations [73]. Across all UC subtypes, FGFR3 mutations are detectable in 20%, and FGFR3 overexpression in 50%, of cases [74]. As part of the BLC2001 study, Erd as an FGFR 1–4 tyrosine kinase inhibitor (TKI) was tested in 22 patients that had received prior CPI therapy. Only 1/22 patients (5%) had achieved any kind of response to prior CPI. The molecular subtypes of the study participants’ cancers were not demonstrated, but at least one FGFR3 mutation or FGFR2/3 fusion was required for study inclusion [44]. FGFR mutations or fusions seem to indicate inferior CPI response. A similar conclusion can be drawn from data of a study with Rogaratinib, another FGFR 1–4 TKI: 90% of patients who had received prior CPI had best response of PD under CPI therapy. The ORR to the study medication was 30% at least [75].

In contrast, basal UC subtypes can be the target of therapy with EGFR inhibitors like RAM [76]. Up to seventy-four percent of bladder cancers exhibit EGFR overexpression [77]. In basal UC subtypes, genes encoding for EGFR or up or downstream ligands or targets of EGFR are overexpressed, and basal UC cell lines are sensitive to EGFR inhibitors [76]. During the RANGE study, the addition of RAM to docetaxel did not improve OS compared to CT alone in the general collective [43], and the ORR of D + R in the subgroup of CPI-pretreated patients was clearly limited (29.4%). Interestingly, a separate molecular analysis of the patients’ tumor biopsies showed an overlap of positive PD-L1 status and basal subtypes. The group of patients with positive PD-L1 status and basal subtype did benefit from the addition of RAM: median OS was 9.2 months for D + R and 6.0 months for *D + P* (*p* = 0.01) [78]. Again, there is the possibility that a subgroup of patients may benefit from therapy with EGFR inhibitors even after progression under CPI therapy.

A signaling cascade close to the EGFR pathway involves another target known from uro-oncology: vascular endothelial growth factor (VEGF). EGFR expression causes VEGF signaling, whereas VEGF upregulation indicates EGFR resistance [79]. VEGF inhibitors are used in the therapy of for example metastatic renal cell carcinoma. Nevertheless, VEGF inhibitors have not yet been able to establish themselves in mUC therapy. Sorafenib, Pazopanib, Cabozantinib, Sunitinib, Vandetanib and Bevacizumab, alone or in combination with CT such as GemCis or Docetaxel, mostly developed toxicities without apparent benefit [80,81,82,83].

In addition to the targets already mentioned, inhibitors of various other pathways and signaling cascades are being investigated in mUC.

Inhibitors of human epidermal growth factor receptor 2 (HER2) are already the standard of care in gynecologic oncology. The data on the rate of HER2 expression in UC varies widely, with rates ranging from 9–72% [84,85,86]. A high expression of HER2 is significantly associated with aggressive disease and poor prognosis [87,88]. New data from a phase II study testing the ADC RC48 (Disitamab Vedotin) targeting HER2 in heavily pretreated HER2 positive mUC was published in October 2020 by Sheng et al. [89]. Of the patients, 18.6% had received CPI therapy before study inclusion. The ORR was 51% in the general collective and 75% in the CPI pretreated patients. A subgroup of patients with HER2 positive mUC may benefit in the future.

Hormone blockade is also a conceivable approach in the treatment of mUC. Thirteen to fifty-three percent of bladder cancer specimens express the androgen receptor (AR), regardless of the patient’s sex [90,91]. The density of the AR correlates negatively with staging and grading of the tumor [92,93], indicating that loss of androgen sensitivity is associated with tumor progression [94]. Enzalutamid as a next-generation AR inhibitor is currently being investigated in combination with platinum-based CT in mUC in a phase I/Ib study [95]. AR blockade may be an option for mUC patients with AR positive tumors.

Another possible target therapy for mUC are poly (ADP-ribose) polymerase (PARP) inhibitors. The PARP-inhibitors target DNA repair gene mutations, which can be found in 30–60% of muscle-invasive UC [96]. The first data from the ATLAS study testing the PARP-inhibitor Rucaparib in patients with mUC after platinum-based CT and/or CPI therapy, were presented at ASCO-GU 2020 [97]. Of the patients, 73.2% (*n* = 97) had received previous CPI therapy. Up to the date of publication, the study medication has failed to achieve any confirmed response, even in the subgroup of hormone receptor positive patients. The median PFS was 1.8 months and the study was discontinued. Several other PARP-inhibitors are currently still being tested alone or in combination with CT, CPI or SG [98] in different therapy settings of UC.

Various mTOR inhibitors such as Everolimus and Sapanisertib are also experiencing a revival in uro-oncology in the treatment of mUC, both alone and in combination with CPI therapy [99,100].

The treatment of mUC has changed considerably in recent years. CT and CPI therapy are now applied almost as standard therapy in the neoadjuvant, adjuvant and palliative setting. Combination therapies of new ADC or target therapies with CT or CPI therapy are being investigated in all settings. Data from the EV-103 study led to another FDA breakthrough therapy designation for EV in combination with Pembrolizumab in cisplatin-ineligible mUC patients [101]. The study showed an ORR of 73.3%, with higher ORR in patients with high PD-L1 status (78.6% vs. 63.2% in PD-L1 low status). The KEYNOTE-905/EV-303 trial is evaluating Pembrolizumab with/without EV in the neoadjuvant setting prior to cystectomy in cisplatin-ineligible patients [102]. The KEYNOTE-B15/EV-304 study is investigating the same combination in the same setting for cisplatin-eligible patients [103]. Another study on the combination therapy of SG and EV in mUC after platinum-based CT and CPI therapy is to start soon [60].

To take us further into the question of sequences and combination therapies, the MORPHEUS-mUC study has certainly an interesting concept [104]. Here, different second-line therapies of mUC are to be combined in order to optimize immune cell activation and target recognition, and thus to potentiate the effect of the therapies. Patients should receive either Atezolizumab mono or in combination with EV, or antibodies against PARP, CD47, CD38, DPP-4 and IL-6R. Especially studies investigating combination therapies, in addition to basic research, will certainly further contribute to understanding and utilizing the complex interplay of the immune system in tumor therapy.

In addition, there is an ever deeper understanding of the diversity of the tumor disease as such. The subdivision of tumor disease into specific subgroups, with different genetics, dynamics and demands on the therapy, enables customized systemic therapies. Increasingly, genome sequencing appears to play a central role in therapy selection [105]. Two of the therapeutic approaches mentioned, Erd and EV, are already on their way to the clinic and have FDA approvals. Since Erd has only been studied and approved in patients with certain FGFR3 mutations, the expression of this characteristic can help in the treatment decision. The newest version of the EAU guidelines on MIBC already addresses this circumstance [106]: in cases of disease progression of mUC under CPI therapy, FGFR3 analysis should be performed. If present, Erd is a possible therapy option beside CT with paclitaxel, docetaxel and Vinflunine. If not, EV can be offered.

The more we understand about molecular mechanisms and the importance of the UC subtypes, the better we can select individualized therapy for our patients. Still, little is known about the complex interplay of PD-1/PD-L1 with other signaling cascades. The development of markers and the identification of therapy targets may become useful, especially in later therapy lines, in order to achieve optimal therapeutic success and not to limit the quality of life with potentially ineffective substances. The question of the therapy sequence is certainly becoming increasingly important. Which therapy can pave the way for another therapy, which therapy can act synergistically with CPI therapy or effectively treat the activation of compensatory growth signals of the tumor?

### Limitations

For better comparability of the outcome data, our working group combined the data of the studies EV-101 (in parts), 201 and 301 in a single-group meta-analysis. Statistically, it has been shown that the data of the EV-301 study partially deviates from the data of the EV-101 and 201 studies. This was considered in using the statistical models. If additional study data is available in the future, a further modification of the data may be possible.

The demographic data of the study participants shown are valid for the entire collective of study participants. In the case of EV, these are also study participants with divergent dosages of EV or without previous CPI therapy. This applies especially to the EV-101 study. In the single-group meta-analysis of the studies testing EV, in the absence of specific demographic data on the subgroup of CPI pretreated patients with a dosage of 1.25 mg EV/kg of body weight, it was assumed that the distribution of the demographic data of the overall group was representative for the subgroup mentioned.

The effect size of time-to-event variables (DoR, PFS, OS) was estimated based on the Kaplan-Meier estimators. It should be noted that the observation periods of the studies differed. In addition, the data were collected once a month in two out of three studies and bi-monthly in one out of three studies. Thus, the above data should be considered approximate.

## 5. Conclusions

The treatment of mUC after progression on CPI therapy represents an upcoming challenge. Currently, few innovative treatment options are available in this setting. Most data are certainly available on EV. In this single-group meta-analysis, the results of *n* = 74 + 125 + 288 study participants under EV were pooled. The response rates of EV are particularly compelling: the ORR of EV was 42.1% vs. 17.9% for CT in a similar setting. EV was also ahead in PFS (5.9 months with EV vs. 3.7 months with CT) and OS (12.8 months with EV vs. 9.0 months with CT), although not directly comparable. The two new agents, Erd and SG, are still under further investigation, and few data are available for the specific setting of CPI-pretreated patients. The tolerability goes in the same direction for all agents: AE rates are 84.2–93.8%, and G3 AEs 40.8–52.3%.

Both the histological and molecular genetic properties of the BC appear to have an impact on the response to CPI and newer therapeutic agents. In the post CPI setting, the EAU guideline on MIBC recommends FGFR3 analysis, and subsequently gives us the option of therapy with EV (status negative) or Erd (status positive) as an alternative to CT with paclitaxel, docetaxel and Vinflunine. The newer agents are currently being investigated in all scenarios of UC, alone or in combinations with each other, or with CPI or CT. In addition to basic research, the findings will certainly further contribute to understanding and utilizing the complex interplay of the immune system in tumor therapy.

## Figures and Tables

**Table 1:** * Paclitaxel, Docetaxel, Vinflunine. ° Selected regimen collective, treated with 8 mg of Erd continuously. ^∆^ Pooled data. ^▀^ Data only available for *n* = 155 [40] + 125 [41]. ^●^ Bladder cancer subgroup, treated with 10 mg SG/kg of body weight.

Medication		CT * [42]	D ± R [43]		Erd ° [44]	EV ^∆^ [40,41,42]	SG ^●^ [45,46]
			D + R	D + P			
Study phase		4	3		2	1, 2, 3	2
Number of patients (*n*)		307	263	267	99	155 + 125 + 301	45
Median age in years (Range)		68 (30–88)	65 (59–72)	66 (59–72)	68 (36–87)	68 (24–86)	67 (49–90)
Sex male, %		75.6	80.9	80.5	76.8	75.2	91.1
ECOG = 0, %		40.4	46.0	46.8	50.5	35.5	31.0
ECOG ≥ 1, %		59.6	52.9	53.2	49.5	64.5	69.0
Sites of metastasis	Visceral, %	81.7	69.2	70.4	78.8	80.2	73.3
	Lung, %	NA	37.3	45.3	57.6	47.1 ^▀^	60.0
	Liver, %	30.9	29.7	25.8	20.2	34.9	33.3
	Bone, %	NA	21.3	19.9	21.2	NA	NA
	Lymph nodes only, %	9.2	15.6	15.7	21.2	10.2	NA
Prior therapies before study inclusion	CT platinum base, %	100.0	NA	98	87.9	99.0	95.0
	CT cisplatinum-base, %	NA	61.2	70.8	NA	74.6 ^▀^	NA
	CT carboplatinum-base, %	NA	36.9	28.8	NA	37.1 ^▀^	NA
	CPI, %	100.0	6.5	10.5	22.2	92.6	38.0

**Table 2:** * Paclitaxel, Docetaxel, Vinflunine. ° Selected regimen collective, treated with 8 mg of Erd continuously. **^∆^** Pooled data, all patients with prior CPI therapy treated with 1.25 mg EV/kg body weight available for response evaluation. ^▼^ Estimated using the plots of Kaplan-Meier. ^π^ *n* = 33 [40] + 55 [41] + 117 [42]. ^▯^ *n* = 74 [40] + 125 [41] + 301 [42]. ^±^ *n* = 89 [40] + 125 [41] + 301 [42].

Medication	CT * [42]	D ± R [43]		Erd ° [44]	EV ^∆^ [40,41,42]	SG [45,46]
		D + R	D + P			
Number of CPI pretreated patients (*n*)	296	17	28	22	74 + 125 + 288	17
ORR (%) (95% CI)	17.9 (13.7; 22.8)	29.4	7.1	59.0	42.1 (37.8; 46.5)	23.5
CR (%) (95% CI)	2.7	0	0	NA	8.5 (4.6; 15.1)	NA
PR (%) (95% CI)	15.2	29.4	7.1	NA	34.5 (30.4; 38.9)	NA
SD (%) (95% CI)	35.5	35.3	57.1	NA	31.3 (27.3; 35.5)	NA
PD (%) (95% CI)	28.0	17.6	25.0	NA	16.7 (13.6; 20.3)	NA
Median DoR in months	8.1 (5.7; 9.6)	NA	NA	NA	7.5 ^▼,π^	NA
Median PFS in months (95% CI)	3.7 (3.5; 3.9)	NA	NA	NA	5.9 (5.4; 6.6) ^▼,▯^	NA
Median OS in months	9.0 (8.1; 10.7)	NA	NA	NA	12.8^, ▼,±^	NA

**Table 3:** All data presented in percentage terms. * Paclitaxel, Docetaxel, Vinflunine. ^●^ pooled data, EV dosage of 1.25 mg/kg of body weight. ^▯^ Data only available for *n* = 125 [41] + 296 [42]. ^▼^ All cancer types, subgroup with SG dosage of 10 mg/kg of body weight.

Medication	CT * [42]	D ± R [43]		Erd [44]	EV ^●^ [40,41,42]	SG ^▼^ [45]
		D + R	D + P			
Number of patients (*n*)	291	258	265	99	112 + 125 + 296	97
Treatment related AE, %	91.8	85.7	84.2	NA	93.8 (91.1–95.8) ^▯^	91.8
Treatment related ≥G3 AE, %	49.8	47.7	40.8	46.0	52.3 (47.5–57.0) ^▯^	NA
Fitness						
Asthenia, %	NA	NA	NA	20.2	NA	NA
≥G3 asthenia, %	NA	NA	NA	7.1	NA	NA
Fatigue, % (95% CI)	22.7	39.1	36.2	32.3	43.9 (29.8–58.9)	47.4
≥G3 fatigue, % (95% CI)	4.5	6.6	6.0	2.0	5.7 (3.9–8.1)	8.2
Skin, hair, nail and mucosa						
Alopecia, % (95% CI)	36.4	23.6	30.6	29.3	46.3 (42.1–50.6)	34.0
≥G3 alopecia, %	0	0	0.4	0	0	NA
Dry mouth, %	NA	NA	NA	45.5	NA	NA
≥G3 dry mouth, %	NA	NA	NA	0	NA	NA
Dry skin, % (95% CI)	NA	NA	NA	32.3	21.9 (17.1–27.7)	NA
≥G3 dry skin, %	NA	NA	NA	0	0	NA
Hand-foot syndrome, %	NA	NA	NA	23.2	NA	NA
≥G3 hand-foot syndrome, %	NA	NA	NA	5.1	NA	NA
Maculopapular rash, % (95% CI)	NA	NA	NA	NA	20.9 (15.2–27.9)	NA
≥G3 maculopapular rash, % (95% CI)	NA	NA	NA	NA	6.0 (4.3–8.5)	NA
Nail dystrophy, %	NA	NA	NA	16.2	NA	NA
≥G3 nail dystrophy, %	NA	NA	NA	6.1	NA	NA
Pruritus, % (95% CI)	NA	NA	NA	NA	27.6 (18.7–38.7)	NA
≥G3 pruritus, % (95% CI)	NA	NA	NA	NA	1.1 (0.5–2.6)	NA
Stomatitis, %	NA	23.3	9.1	57.6	NA	NA
≥G3 stomatitis, %	NA	3.5	0	10.1	NA	NA
Gastrointestinal and urinary tract						
Abdominal pain, %	NA	NA	NA	NA	NA	22.7
≥G3 abdominal pain, %	NA	NA	NA	NA	NA	3.1
Constipation, %	NA	NA	NA	28.3	NA	30.9
≥G3 constipation, %	NA	NA	NA	1.0	NA	1.0
Decreased appetite/anorexia, % (95% CI)	23.4	22.1	17.0	38.4	38.3 (29.6–47.7)	NA
≥G3 decreased appetite/anorexia, %	1.7	1.6	0.4	0	2.4 (1.3–4.3)	NA
Diarrhoea, % (95% CI)	NA	23.6	16.6	50.5	28.1 (24.5–32.1)	56.7
≥G3 diarrhoea, % (95% CI)	NA	3.1	1.1	4.0	2.9 (1.7–4.8)	9.3
Dysgeusia, % (95% CI)	NA	NA	NA	37.4	33.5 (23.4–45.4)	NA
≥G3 dysgeusia, %	NA	NA	NA	1.0	0	NA
Nausea, % (95% CI)	21.6	22.1	14.0	20.2	32.3 (21.8–45.0)	57.7
≥G3 nausea, % (95% CI)	1.4	0.8	0.8	1.0	1.4 (0.7–3.0)	2.1
UTI, %	NA	NA	NA	16.2	NA	NA
≥G3 UTI, %	NA	NA	NA	5.1	NA	NA
Vomiting, %	NA	NA	NA	NA	NA	39.2
≥G3 vomiting, %	NA	NA	NA	NA	NA	3.1
Laboratory changes						
Anemia, %	20.3	11.6	16.2	20.2	NA	38.1
≥G3 anemia, %	7.6	1.9	5.3	4.0	NA	11.3
Hyponatremia, %	NA	NA	NA	12.1	NA	NA
≥G3 hyponatremia, %	NA	NA	NA	11.1	NA	NA
Hyperphosphatemia, %	NA	NA	NA	76.8	NA	NA
≥G3 hyperphosphatemia, %	NA	NA	NA	2.0	NA	NA
Leucopenia, %	NA	NA	NA	NA	NA	NA
≥G3 leucopenia, %	NA	NA	NA	NA	NA	NA
White blood cell count decrease, %	10.7	6.6	7.5	NA	NA	17.5
≥G3 White blood cell count decrease, %	6.9	4.3	6.4	NA	NA	11.3
Neutrophil count decrease, %	16.8	11.6	10.6	NA	10.2 (7.7–13.5)	53.6
≥G3 Neutrophil count decrease, %	13.4	8.9	10.2	NA	6.7 (4.7–9.5)	33.0
Neutropenia, %	8.2	8.5	4.2	NA	NA	NA
≥G3 Neutropenia, %	6.2	6.6	2.3	NA	NA	NA
Neutropenia, febrile, %	5.5	9.3	6.0	NA	NA	NA
≥G3 neutropenia, febrile, %	5.5	9.3	6.0	NA	NA	6.2
Others						
PSN, % (95% CI)	21.3	NA	NA	NA	36.1 (32.1–40.2)	NA
≥G3 PSN, % (95% CI)	2.1	NA	NA	NA	2.5 (1.4–4.3)	NA

## Data Availability

Part of the data is available in the Supplementary Material, part on request from the authors.

## Supplementary Materials

The following are available online at https://www.mdpi.com/article/10.3390/cancers13133206/s1, File S1, presentation of selected statistical calculations of the single group meta-analysis of three studies testing Enfortumab vedotin.

## Abbreviations

ADC	Antibody-drug conjugate
AEs	Adverse events
G3 AE	Grade 3 adverse event
AKT	Protein kinase B
AR	Androgen receptor
ASCO-GU	Genitourinary Cancers Symposium of the American Society of Clinical Oncology
BC	Bladder cancer
NMIBC	Nonmuscle invasive bladder cancer
MIBC	Muscle invasive bladder cancer
BSC	Best supportive care
CPI	Checkpoint inhibition
CR	Complete response
CT	Chemotherapy
D + R/P	Docetaxel + Ramucirumab/Placebo
DoR	Duration of response
EAU	European Association of Urology
ECOG	Eastern Cooperative Oncology Group
EGFR	Epidermal Growth Factor Receptor
EMA	European Medicines Agency
Erd	Erdafitinib
ESMO	European Society for Medical Oncology
ESMO	European Society for Medical Oncology
EV	Enfortumab vedotin
FDA	United States Food and Drug Administration
FGFR	Fibroblast growth factor receptor
FU	Follow up
GA	Genetic alterations
HER2	Human epidermal growth factor receptor 2
i.v.	Intravenously
IgG	Immunglobulin G
ITT	Intention to treat
MAPK	Mitogen-activated protein kinase
MHC	Major histocompatibility complex
mTOR	Mechanistic Target of Rapamycin
M-VAC	Methotrexate, Vinblastine, Adriamycin and Cisplatin
NA	Not available
NR	Not reached
ORR	Objective response rate
OS	Overall survival
PARP	poly(ADP-ribose) polymerase
PD	Progressive disease
PD-(L)1	Programmed cell death (ligand)-1
PFS	Progression free survival
PR	Partial response
PRISMA	Preferred Reporting Items for Systematic Review and meta-analysis Statement
PSN	Peripheral sensory neuropathy
RAM	Ramucirumab
RECIST	Response evaluation criteria in solid tumors
RTK	Receptor tyrosine kinases
SG	Sacituzumab govitecan
SRM	Selected regimen
TCGA	The Cancer Genome Atlas
TKI	Tyrosine kinase inhibitor
(m)UC	(Metastatic) urothelial carcinoma
UTI	Urinary tract infection
VEGF	Vascular endothelial growth factor
WBC	White blood cell
