# Supplementary material for: Current Systemic Treatment Options in Metastatic Urothelial Carcinoma after Progression on Checkpoint Inhibition Therapy—A Systemic Review Combined with Single-Group Meta-Analysis of Three Studies Testing Enfortumab Vedotin
Source: Cancers (Basel). 2021 Jun 26;13(13):3206. doi: 10.3390/cancers13133206 (PMC8268971; doi:10.3390/cancers13133206)
Supplement: Supplementary file 1 [file cancers-13-03206-s001.zip › cancers-1222385-supplementary.pdf]

cancers

MDPI

Supplementary material

Current Systemic Treatment Options in Metastatic Urothelial Carcinoma after Progression on Checkpoint Inhibition Therapy—A Systemic Review Combined with Single-Group Meta-Analysis of Three Studies Testing Enfortumab Vedotin

Susanne Deininger, Peter Törzsök, David Oswald and Lukas Lusuardi

Presentation of selected statistical calculations of the single group meta-analysis of three studies testing Enfortumab vedotin (EV) in a dosage of 1.25 mg/ kg of body weight in metastatic urothelial cancer after progression under platinum- based chemotherapy (CT) and checkpoint inhibitor (CPI) therapy [1-3]. The following numbering applies to the entire document: study 1 [1], study 2 [2] and study 3 [3].

1. Response rates

1.1. Confirmed complete response (CR)

	study 1	study 2	study 3	total
n_{meta}	74	125	288	487
n_{event}	8	15	14	37

Estimated heterogeneity:

- $Tau^2 = 0.24$
- Higgins $I^2 = 72.1\%$
- Cochran's $Q_{het} = 7.18$ (p = **0.028***)

Statistically relevant heterogeneity between the studies was evident, thus the random effect model was used for the present data situation.

Random effect model:

Study	Effect estimators	95%-Confidence Interval (CI)		Weight of the study
		Lower limit	Upper limit	
1	10.81%	4.78%	20.20%	29.3%
2	12.00%	6.87%	19.02%	35.3%
3	4.86%	2.68%	8.02%	35.4%
Meta effect estimators	8.52%	4.62%	15.17%	100%

Forest plot:

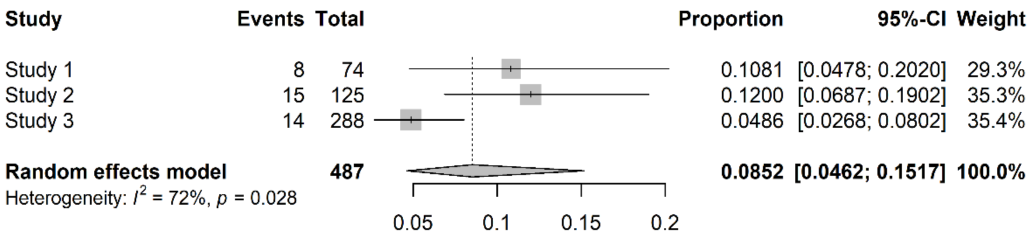

1.2. Confirmed partial response (PR)

	study 1	study 2	study 3	total
n_{meta}	74	125	288	487
n_{event}	25	40	103	168

Estimated heterogeneity:

- $Tau^2 = 0$
- Higgins $I^2 = 0\%$
- Cochran's $Q_{het} = 0.57$ ($p = 0.754$)

There was no statistically relevant heterogeneity between the studies, thus the fixed effect model was used for the present data situation.

Fixed effect model:

Study	Effect estimators	95%-Confidence Interval (CI)		Weight of the study
		Lower limit	Upper limit	
1	33.78%	23.19%	45.72%	15.1%
2	32.00%	23.94%	40.93%	24.7%
3	35.76%	30.23%	41.60%	60.2%
Meta effect estimators	34.52%	30.42%	38.85%	100%

Forest: plot:

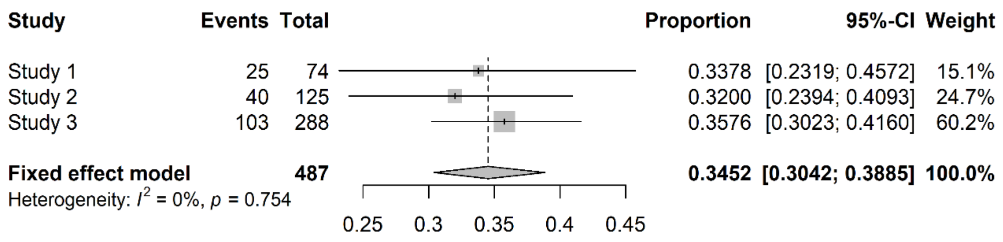

1.3. Confirmed stable disease (SD)

	study 1	study 2	study 3	total
n_{meta}	74	125	288	487
n_{event}	27	35	90	152

Estimated heterogeneity:

- $Tau^2 = 0$
- Higgins $I^2 = 0\%$
- Cochran's $Q_{het} = 1.55$ ($p = 0.460$)

There was no statistically relevant heterogeneity between the studies, thus the fixed effect-model was used for the present data situation.

Fixed effect model:

Study	Effect estimators	95%-Confidence Interval (CI)		Weight of the study
		Lower limit	Upper limit	
**1**	**36.49%**	25.60%	48.49%	16.5%
2	28.00%	20.34%	36.73%	24.2%
3	31.25%	25.94%	36.95%	59.4%
Meta effect estimators	31.27%	27.30%	35.53%	100%

Forest plot:

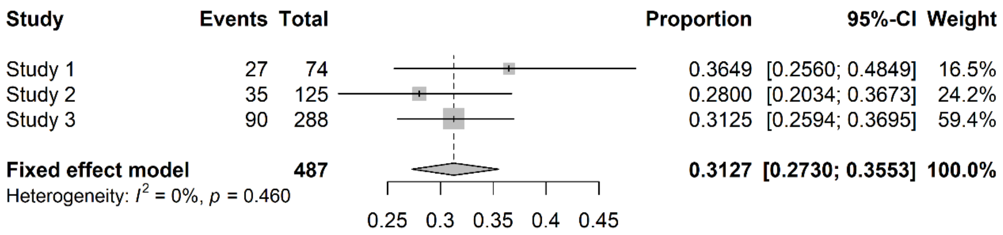

1.4. Confirmed progressive disease (PD)

	study 1	study 2	study 3	total
n_{meta}	74	125	288	487
n_{event}	14	23	44	81

Estimated heterogeneity:

- $Tau^2 = 0$
- Higgins $I^2 = 0\%$
- Cochran's $Q_{het} = 0.94$ ($p = 0.625$)

There was no statistically relevant heterogeneity between the studies, thus the fixed effect model was used for the present data situation.

Fixed effect model:

Study	Effect estimators	95%-Confidence Interval (CI)		Weight of the study
		Lower limit	Upper limit	
1	18.92%	10.75%	29.70%	16.8%
2	18.40%	12.04%	26.32%	27.8%
3	15.28%	11.33%	19.96%	55.3%
Meta effect estimators	16.70%	13.63%	20.28%	100%

Forest plot:

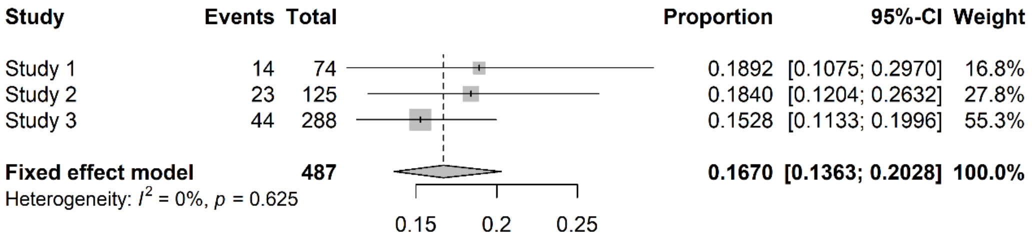

1.5. Objective response rate (ORR)

	study 1	study 2	study 3	total
n_{meta}	74	125	288	487
n_{event}	33	55	117	205

Estimated heterogeneity:

- $Tau^2 = 0$
- Higgins $I^2 = 0\%$
- Cochran's $Q_{het} = 0.63$ ($p = 0.730$)

There was no statistically relevant heterogeneity between the studies, thus the fixed effect model was used for the present data situation.

Fixed effect model:

Study	Effect estimators	95%-Confidence Interval (CI)		Weight of the study
		Lower limit	Upper limit	
1	44.59%	33.02%	56.61%	15.4%
2	44.00%	35.14%	53.16%	26.0%
3	40.62%	34.90%	46.54%	58.6%
Meta effect estimators	42.10%	37.79%	46.54%	100%

Forest plot:

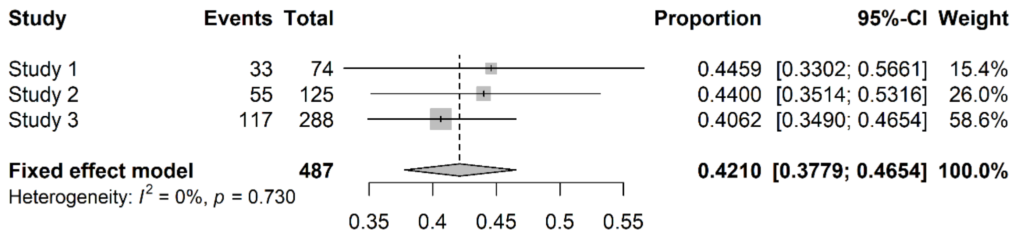

2. Time- to- event variables

2.1. Duration of response (DoR)

	study 1	study 2	study 3
n_{meta}	33	55	117
n_{event}	7.5	7.6	7.39

Effect estimators of the single-group meta-analysis based on the plots of Kaplan Meier [4]:

	Effects estimator	95%- CI
Meta effect estimator median DoR	7.48	not predictable

Estimated heterogeneity: Higgins I^2 = 31.72%.

2.2. progression free survival (PFS)

	study 1	study 2	study 3
n_{meta}	74	125	301
n_{event}	6.6	5.8	5.55

Effect estimators of the single-group meta-analysis based on the plots of Kaplan Meier [4]:

	Effect Estimator	95%- CI	
		Lower limit	Upper limit
Meta effect estimator median PFS	5.93	5.41	6.57

Estimated heterogeneity: Higgins I^2 = 0%.

2.3. overall survival (OS)

	study 1	study 2	study 3
n_{meta}	89	125	301
n_{event}	12.3	11.7	12.88

Effect estimators of the single-group meta-analysis based on the plots of Kaplan Meier [4]:

	Effect estimator	95%- CI
Meta effect estimator median OS	12.81	not predictable

Estimated heterogeneity: Higgins $I^2 = 5.2\%$.

Reference

1. Rosenberg, J.; Sridhar, S.S.; Zhang, J.; Smith, D.; Ruether, D.; Flaig, T.W.; Baranda, J.; Lang, J.; Plimack, E.R.; Sangha, R.; et al. EV-101: A Phase I Study of Single-Agent Enfortumab Vedotin in Patients With Nectin-4-Positive Solid Tumors, Including Metastatic Urothelial Carcinoma. *J. Clin. Oncol.* **2020**, *38*, 1041-1049, doi:10.1200/JCO.19.02044.
2. Rosenberg, J.E.; O'Donnell, P.H.; Balar, A.V.; McGregor, B.A.; Heath, E.I.; Yu, E.Y.; Galsky, M.D.; Hahn, N.M.; Gartner, E.M.; Pinelli, J.M.; et al. Pivotal Study of Enfortumab Vedotin in Urothelial Carcinoma After Platinum and Anti-Programmed Death 1/Programmed Death Ligand 1 Therapy. *J Clin. Oncol.* **2019**, *37*, 2592-2600, doi:10.1200/JCO.19.01140.
3. Powles, T.; Rosenberg, J.E.; Sonpavde, G.P.; Loriot, Y.; Duran, I.; Lee, J.L.; Matsubara, N.; Vulsteke, C.; Castellano, D.; Wu, C.; et al. Enfortumab Vedotin in Previously Treated Advanced Urothelial Carcinoma. *N. Engl. J. Med.* **2021**, *384*, 1125-1135, doi:10.1056/NEJMoa2035807.
4. Combescure, C.; Foucher, Y.; Jackson, D. Meta-analysis of single-arm survival studies: a distribution-free approach for estimating summary survival curves with random effects. *Stat. Med.* **2014**, *33*, 2521-2537.
